# Editorial: Neuromechanics of Hip Osteoarthritis

**DOI:** 10.3389/fspor.2021.788263

**Published:** 2021-11-10

**Authors:** Laura E. Diamond, Rod S. Barrett, Luca Modenese, Andrew E. Anderson, Michelle Hall

**Affiliations:** ^1^Griffith Centre of Biomedical and Rehabilitation Engineering, Menzies Health Institute Queensland, Griffith University, Gold Coast, QLD, Australia; ^2^School of Health Sciences and Social Work, Griffith University, Gold Coast, QLD, Australia; ^3^Department of Civil and Environmental Engineering, Imperial College London, London, United Kingdom; ^4^University of Utah Motion Capture Core Facility, University of Utah, Salt Lake City, UT, United States; ^5^Centre for Health, Exercise and Sports Medicine, The University of Melbourne, Melbourne, VIC, Australia

**Keywords:** biomechanics, mechanical load, hip OA, femoroacetabular impingement syndrome, dysplasia

## Introduction

Hip osteoarthritis (OA) adversely affects the quality of life of millions of people worldwide and imposes a substantial burden on related healthcare systems (Murphy et al., 2010). Rates of total hip replacement are escalating, highlighting the urgent need to improve evidence-based non-surgical treatments for people with hip OA. Joint replacement surgery is costly and reserved for end-stage disease when non-surgical treatments are no longer effective (Katz et al., 2021). Approximately 60% of people with hip OA undergo joint replacement within 3 years of first presenting to a healthcare professional (Dabare et al., 2017). This timeline highlights a relatively short window of opportunity for effective non-surgical intervention, and the importance of investigations of people at risk for development of hip OA (e.g., femoroacetabular impingement syndrome, developmental dysplasia of the hip). Clinical guidelines for OA emphasise that non-drug and non-surgical treatments are core management strategies for hip OA (Bannuru et al., 2019; Kolasinski et al., 2020). Nevertheless, these conservative management strategies typically only provide small-to-modest improvements in pain, function, and quality of life. Poor outcomes following conservative management may in part be due to the fact that targets for treatment are not well-defined and not personalised to the patient.

## Altered Neuromechanics Across the Spectrum of Disease

Sub-optimal mechanical loading is believed to play a critical role in hip OA progression, and may be modifiable through a range of approaches including exercise and movement retraining. Hip loads interact with cartilage mechanobiology (i.e., tissue mechanics) (Pizzolato et al., 2017) to regulate cartilage structure and have potential to contribute to or regulate osteoarthritic processes, including neuro-immune responses that underpin the transition from early OA to late stage disease (Ren and Dubner, 2010). In early OA, patients typically report pain during weight-bearing activities (e.g., negotiating stairs), whereas patients with advanced OA tend to report more widespread pain that often occurs at rest (Neogi, 2013). Characterising the mechanical environment of the hip is an important, yet challenging task. Identifying alterations in neuromechanical factors, including movement patterns, muscle activation patterns, skeletal and joint geometry, and muscle-tendon morphology, and their complex interactions is critical for establishing clearly defined treatment targets.

Altered muscle forces and movement patterns during activities of daily living, such as walking and stair climbing, are thought to be responsible for abnormal hip loading in hip OA. Lewis et al. showed that kinematic alterations oberved in people with established hip OA present much earlier in the disease course – in those with painful intra-articular hip conditions (i.e., diagnosed femoroacetabular impingement syndrome, developmental dysplasia of the hip, or labral tear) widely considered precursors for the development of hip OA. Notably, the lower hip flexion reported during walking in this cohort compared to controls could potentially alter hip loading–including magnitude, distribution, and location within the acetabulum. Looking specifically at participants with hip dysplasia, Song et al. used musculoskeletal modelling, including personalised hip geometry and muscle paths, to show higher edge loading in the antero-superior acetabulum during walking compared to controls. Taken together, these studies support the notion that an altered mechanical environment is not only present in people with established hip OA (Meyer et al., 2018; Diamond et al., 2020), but also in those at risk for disease development, and is highly dependent on movement, muscle, and joint geometry. The extent to which mechanical targets are modifiable (van Veen et al., 2019) and capable of slowing disease progression and improving long term functional outcomes remains an open area of investigaton and an important directive for furture research.

## Effect of Exercise on Neuromechanics of The Hip

Exercise is a core evidence-based treatment recommended for hip OA across all current clinical guidelines (Fernandes et al., 2013; National Clinical Guideline Centre, 2014; Bannuru et al., 2019; Kolasinski et al., 2020). The beneficial effects of exercise on pain and physical function are modest at best (Fransen et al., 2014), with evidence from a meta-analysis highlighting the importance of prescribing exercise dose in accordance with recommended guidelines to attain greater clinical benefits (Moseng et al., 2017, 2018). Of note, all trials in the review included muscle strengthening, while few trials included aerobic exercise (Moseng et al., 2017). Indeed, the majority (95%) of physiotherapists prescribe muscle strengthening exercises for people with hip OA (Cowan et al., 2010). Given the emphasis of exercise for prevention (Kemp et al., 2020) and management of hip OA (Bannuru et al., 2019; Kolasinski et al., 2020), there is as urgent need to better understand the effect of commonly prescribed exercises on hip neuromechanics. The study by Catelli et al. suggested that symptoms and muscle contraction strategy during a bilateral deep squat, rather than bony geometry (i.e., cam morphology) alone, may be the origin of mobility restriction in males with femoroacetabular impingement syndrome. Weightbearing exercises, including squats, are commonly prescribed in the prevention and management of hip OA, and a personalised neuromuscular approach to task execution may be beneficial. Buehler et al. demonstrated that adding an elastic resistance band during hip exercises increased muscle and contact forces, though the values remained below those during walking. The type of elastic resistance band and the velocity at which exercises are executed were also shown to have little impact on hip muscle force generation or hip contact force. Collectively, observations from these hypothesis-generating studies provide helpful preliminary evidence, to be used in conjunction with findings from future studies, to underpin the design of personalised exercise-based interventions for those at risk for and with established hip OA.

## Advanced Approaches for Measurement and Treatment

Use of new and innovative integrated technologies will be pivotal for us to achieve significant gains in our understanding of neuromechanical function in people with hip OA, and the design of efficacious non-surgical interventions. The need to shift neuromechanical measurements to the real-world is evident, given this is where habitual motor control strategies dominate and are most difficult to modify. Recent advances in wearable sensors make them a promising alternative to laboratory-based measurement systems for estimating movement patterns (Slade et al., 2021). While low-cost and easy-to-use, wearable sensors alone cannot assess mechanical loading. However, combining wearable sensor data with other advanced methods, including big data and machine learning (Johnson et al., 2019, 2021; Saxby et al., 2020), biofeedback systems (Pizzolato et al., 2017), and augmented reality (Stanev et al., 2021), has great potential to lead to new understanding of neuromechanical function in people hip OA, and will lend itself to the development of innovative, personalised, efficacious, real-word interventions for management of hip OA.

## Author Contributions

LD and MH wrote the first draught of the editorial. All authors contributed to editorial revision, read, and approved the submitted version.

## Conflict of Interest

The authors declare that the research was conducted in the absence of any commercial or financial relationships that could be construed as a potential conflict of interest.

## Publisher's Note

All claims expressed in this article are solely those of the authors and do not necessarily represent those of their affiliated organizations, or those of the publisher, the editors and the reviewers. Any product that may be evaluated in this article, or claim that may be made by its manufacturer, is not guaranteed or endorsed by the publisher.
